# A Critical Analysis on the Current Design Criteria for Cathodic Protection of Ships and Superyachts

**DOI:** 10.3390/ma15072645

**Published:** 2022-04-04

**Authors:** Davide Clematis, Alessandro Marroccu, Marco Panizza, Antonio Barbucci

**Affiliations:** 1 Department of Civil, Chemical and Environmental Engineering, University of Genova, 16145 Genova, Italy; marco.panizza@unige.it (M.P.); barbucci@unige.it (A.B.); 2 Italy Operational Yachting Marine Surveyor, RINA Services S.p.A., Via Corsica 12, 16128 Genova, Italy; alessandro.marroccu@rina.org; 3 Institute of Condensed Matter Chemistry and Technology for Energy (ICMATE), National Research Council (CNR), c/o DICCA-UNIGE, Via All’Opera Pia 15, 16145 Genova, Italy

**Keywords:** cathodic protection, breakdown factor, numerical simulation, zinc, steel

## Abstract

Classification Society and ISO standard regulate the design of cathodic protection (CP) plans of ships and superyachts. However, due to shipyards’ long experience, the hull vessel protection plans often rely on an adaptation of previous CP designs for similar ships. This simple practice could expose ships to low protection or overprotection. Here, the protection plan of an existing 42 m superyacht is considered to highlight critical CP design issues. The numerical analysis gives evidence of discrepancies between the CP design proposed in accordance with ISO standard and the protection plan that was actually implemented. Indeed, for a proper protection plan, the anode weight according to the ISO standard is 2.7 kg, whereas the real protection plan uses a 7 kg anode. The numerical optimization highlights an optimal anode mass of 5 kg (−28.5% in weight). It provides sufficient protection for the expected lifetime, and will preserve the system in cases of damage to the hull and a consequent increase in the breakdown factor. This new solution underlines the importance and necessity of improving cathodic protection plan design.

## 1. Introduction

Cathodic protection (CP) is a very important method to prevent corrosion of metallic structures [1,2,3,4]. In the case of ships, the protection of the metallic hull is obtained by a combination of an organic coating cycle with a cathodic protection system. Many works underlined the essential role of the coating to protect ship hulls from corrosion [5,6,7,8], and the cathodic protection would be useless if the coating were free of defects, but this is far from being close to reality. Thus, the design and application of a cathodic protection system has to be implemented to guarantee adequate corrosion prevention of the submerged metallic structure. Nevertheless, many efforts are still necessary for improving the protection efficiency of marine ships [9].

Once the metal is exposed to the corrosive media, cathodic protection acts to displace the unavoidable metallic anodic dissolution reaction (1) toward a dedicated anode, leaving the structure to be protected free from oxidation and only involved in the cathodic reaction. See (2) for the oxygen reduction.

(1) $$M\to M^{z+}+ze^{-}$$

(2) $$H_{2}O+\frac{1}{2}O_{2}+2e^{-}\to 2OH^{-}$$

This result is achieved, lowering the hull’s electrode potential toward the cathodic region till an acceptable value is reached. This polarization is obtained through the circulation of a proper current between the ship hull and an auxiliary electrode—namely, the anode. Such an electrode is usually an alloy, and its formulation has to be carefully designed in order to: (i) be sufficiently active and have a low enough electrode potential, (ii) be free from the risk of surface passivity, (iii) have limited autocorrosion, (iv) have an acceptable current capacity expressed in Ah/kg, and (v) be suitable for the material structure’s protection [10,11]. Several materials have been tested for CP applications and showed positive results. Among them, aluminum alloys, such as Zn-Al-Cd and Al-Zn-I, provide acceptable performances, as widely reported in the literature [9]. The Al-based alloys have higher capacities (up to 2400 Ah/kg) than Zn-based alloys (780 Ah/kg).

**Figure 1:**
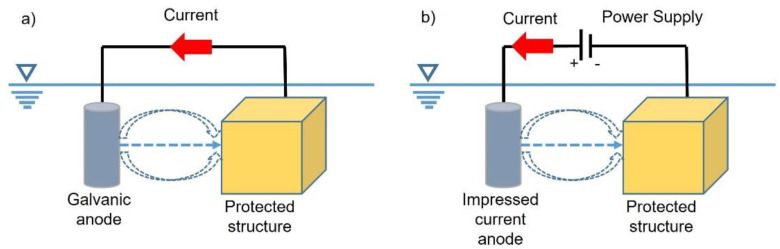
Cathodic protection on ships: (**a**) by galvanic anodes and (**b**) by impressed currents.

The hull’s oxidation reaction can be shifted toward a dedicated anode by galvanic anodes or impressed current, as schematically shown in Figure 1.

In both cases, the ship hull acts as the zone for the cathodic oxygen reaction and its potential is moved to a lower value. In the galvanic anode configuration, the necessary current spontaneously flows, as in a battery. The anode is permanent in the impressed current configuration and hosts the anodic reaction, chloride’s oxidation in seawater. This system does not run spontaneously, and the power supply provides the necessary external work.

The choice of the CP configuration depends on several aspects, but the robustness and simplicity of the galvanic anode concept is an advantage with respect to the impressed current cathodic protection (ICCP) method. On the other hand, the impressed current is a smart and flexible solution that can be adjusted according to external conditions (water conductivity, ship speed, etc.). Moreover, in big ships, CP by impressed current drastically reduces the number of galvanic anodes with unquestionable advantages. Recently, Kalovelonis et al. [12] investigated the design of an ICCP system for a 399 m in length container ship using a boundary element method (BEM). The aim of the authors was to minimize the electric power released by the protection system. However, in this case, galvanic anodes had to always be implemented in the corrosion protection plan to adequately protect zones where the current lines of the impressed current system were disturbed by geometric factors or water turbulence. Therefore, the determination of adequate numbers of anodes and their location is extremely relevant to providing reliable protection while avoiding overprotection. Another approach adopted in ICCP design for ships is physical scale modelling [13]. Wang et al. used a 1/100 scale ship model to evaluate the effectiveness of the proposed ICCP plan and to evaluate the interference due to the external current sources.

As regards the design of a CP plan with a galvanic anode, the regulatory framework is nowadays represented by the International Standard ISO 16222:2012 [14] and the recommended practice (RP) from DNV-RP.B401:2010 [15]. They require that the electric potential for mild steel in seawater to be lower than −0.8 V against Ag/AgCl.

While the DNV-recommended practice mainly refers to offshore platforms, and more generally, to fixed platforms with long lifetimes, the ISO standard proposes a direct approach for naval hulls. The methods proposed by the two regulations are similar, though there are differences in estimating some critical parameters.

Nevertheless, an in-field analysis of real CP plans applied by shipyards highlighted a discrepancy in CP designs based on the international standard. This gap derives from the fact that practice is based on shipyards’ long experience, and their hull vessel protection plans often rely on adaptations of previous CP designs for similar ships. This simple practice could expose ships to low protection or overprotection. In the first case, the potential is too low to protect the whole surface from corrosion, and the second condition leads to high hydrogen production on the cathode surface (ship hull), increasing the formation of blistering or disbonding of the organic coating [16]. Moreover, overprotection causes a waste of the anode material due to faster consumption correlated with higher current circulating in the system.

The development of tools for accurate evaluations of existing CP plans, their optimization, and the design of new ones, is relevant.

Based on the previous points, we performed the research herein to open a new discussion about the design of a cathodic protection plan. Firstly, the anode mass was evaluated according to the ISO standard and compared with those mounted on the hull of a real 42 m superyacht considered as a case study. In the second section of the paper, a numerical simulation is used to critically analyze both cases and to propose an optimization of the anodic weight, confirming the importance of coupling experimental and modelling approaches, as recently reported in the literature [17]. The results show a window of intervention for the improvement of CP layout. Moreover, the proposed method has been developed to be easily transferrable for the design of tailored cathodic protection plans for other applications, such as floating facilities, ballast tanks, and components used in marine environments.

## 2. Materials and Methods

### 2.1. Introduction to the Methodology

**Figure 2:**
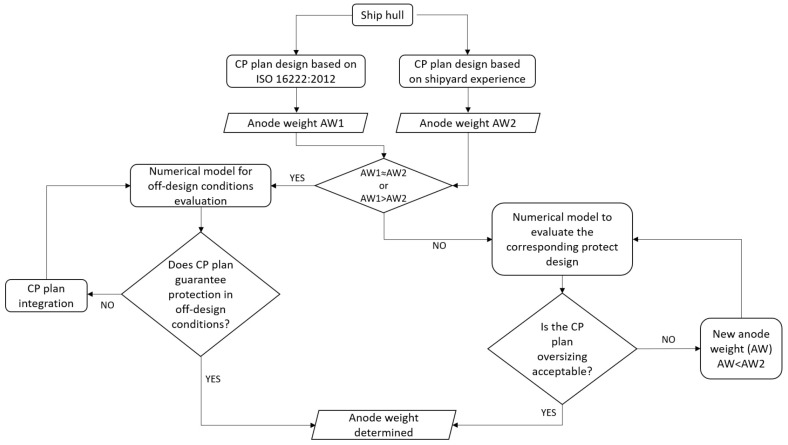
Flowchart of the proposed methodology.

The methodology proposed in this paper was created to provide a tool for evaluating the reliability of a CP plan, and to improve its design. The main steps of the approach are reported in the flowchart in Figure 2, and are described in the following points:

- International Standard ISO 16222:2012 is considered as reference for the design of the CP plans. In particular, the ISO standard is used to calculate the minimum total weight of the anode material needed for protection.
- The obtained results are compared with the real plan adopted by the shipyard, and a numerical model is developed to test the reliability of both solutions and identify critical situations for protection.
- A numerical simulation is used to suggest improvements in CP design.

The method was applied to a case study of a 40 m superyacht, but its structure makes it transferable to a wide range of applications, even for the design of new CP plans.

### 2.2. Regulation for CP on Hulls—ISO Standard

The ISO standard prescribes that the minimum total weight *W_total_* (kg) of the anode material required for a cathodic protection zone has to be calculated from [14]:

(3) $$W_{total}=\frac{I_{c}\times t_{design}\times 8760}{Q\times FU}$$

where:

- *I_c_* is the total reference protection current required for the coated structures [A];
- *t_design_* is the period between dry-docking for the anode system (years);
- *FU* is the utilization factor determined by the portion of the overall anodic material weight to be consumed during the *t_design_*;
- *Q* is the practical anode capacity for the anode material in the environment, and it is a characteristic of the anode itself (Ah/kg).

As regards ships, it has to be taken into account that they experience both dynamic and static conditions depending on the sailing time, and the *I_c_* current, following the ISO standard, can be calculated as [14]:

(4) $$I_{c}=S\times f_{c\;}\times \left(\chi J_{bd}+\left(1-\chi \right)J_{bs}\right)$$

where *S* is the area of the submerged zone that must be protected, *J_bd_* and *J_bs_* are the protection current densities of the bare metal during the navigation and static phase, respectively, and *χ* corresponds to the time fractions in the two different situations. As regards *f_c_*, it is the coating breakdown factor in the period *t_design_*. It considers the percentage of the hull surface that, for whatever reasons, is not covered by the protective coatings and is exposed to the aggressive environment.

The main differences between the ISO standard and the DNV RP are in the estimation of the parameters *J_b_*, *f_c_*, and *FU*, as reported in Table 1.

The current density *J_b_* according to the ISO standard can be assumed as:

- *J_b_* = 100 mA/m^2^ for near static units, such as boats and ships at anchor or berthed (speed v ≤ 1 m/s);
- *J_b_* = 250 mA/m^2^ for fishing ships, utility ships, or units in non-continuous use; (1 < v < 3 m/s)
- *J_b_* = 500 mA/m^2^ for moving vessels (v ≥ 3 m/s).

The breakdown factor, *f_c_*, according to the ISO standard, has to be intended as an initial factor from 1 to 2%. According to EN ISO 12944-1 Im2, an annual increase is considered (from 0.5% to 3%) to be a function of the quality of the organic coating applied to the hull.

### 2.3. Numerical Simulation—Electrochemical Background

The final stage of this research has been devoted to the numerical analysis, using COMSOL^®^ Multiphysics (version5.2, Sweden), of the current and voltage distribution on the electrodes (yacht hull and sacrificial anode) and electrolyte (seawater). First of all, it is crucial to consider that the current and voltage distribution are strictly related to several factors, such as system geometry, material properties, electrode kinetics, and diffusion of the species involved. Nevertheless, with appropriate assumptions, it is possible to simplify the description of the electrochemical system.

The modelling starts assuming that Ohm’s law governs the current and electric potential distribution both in electrodes and electrolyte [18]:

(5) $$i_{e}=-\sigma_{e}\nabla \varphi_{e}$$

(6) $$i_{i}=-\sigma_{i}\nabla \varphi_{i}$$

where *σ* and *φ* are the conductivity and electric potential, respectively, in the electrodes (*e*) and electrolyte (*i*).

The second assumption concerns the electrode kinetics, which determines the distribution of current *i* and then affects the overpotential at the electrode–electrolyte interface. Such relation is described by Butler–Volmer Equation (9) [18]:

(7) i=i++i−=i0eαzFηRT−e−1−αzFηRT

In the case under study, Equation (9) can be simplified because of the polarization region we are most interested in. In fact, for overpotential higher than 0.2 V, it is possible to simplify the terms into the bracket by neglecting the cathodic contribution to the overall current when anode dissolution is considered, or the anodic contribution to the overall current for the oxygen reduction.

Then, two new versions of Equation (9) adapted for reactions (1) and (2) in the logarithmic form are obtained. These Equations (10) and (11) are known as “Tafel equations” and can be applied to the metal oxidation and oxygen reduction reactions, respectively [18]:

(8) $$\eta_{a}=-\frac{RT}{\alpha zF}lni_{0}+\frac{RT}{\alpha zF}lni_{a}\;\;\mathrm{hence}\;i_{a}=i_{0}10^{\frac{\eta }{a}}\;$$

(9) $$\eta_{c}=\frac{RT}{\left(1-\alpha \right)zF}lni_{0}-\frac{RT}{\left(1-\alpha \right)zF}lni_{c}\;\;\mathrm{hence}\;i_{b}=i_{0}10^{\frac{\eta }{b}}$$

where *a* and *b* are the anodic and cathodic Tafel coefficients.

The overpotential provides the connection between electrode and electrolyte physics at the interface defined as [18]:

(10) $$E_{i}=E_{eq}-\varphi_{e}-\varphi_{i}-\left(\eta_{a}+\eta_{c}\right)$$

Moreover, at the boundary between electrode and electrolyte, the following current balance relationship is valid [17]:

(11) $$-i_{e}\cdot n=i_{i}\cdot n$$

with ***n*** being a vector normal to the surface.

The boundary conditions adopted here express the absence of externally generated current density both in electrodes and electrolyte [17]:

(12) $$\varphi_{e,\;ext}=0$$

(13) $$\varphi_{i,ext}=0$$

### 2.4. The Case Study

**Figure 3:**
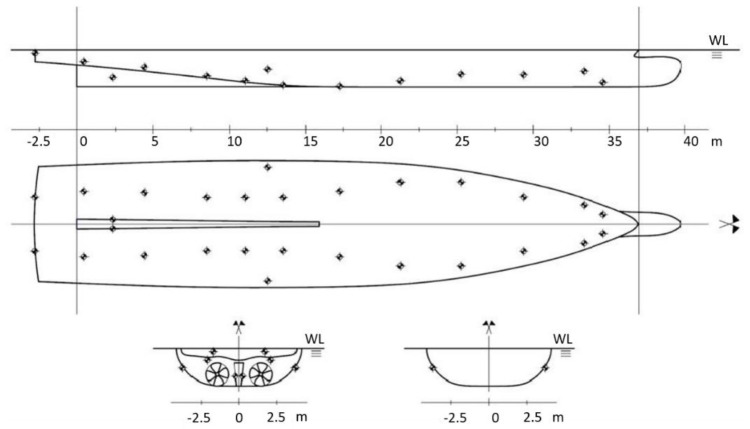
Positioning of galvanic anodes onboard the superyacht.

A steel superyacht already built with its own galvanic anode protection plan is considered as a case study for this analysis and methodology validation. Table 2 and Table 3 and Figure 3 report the geometrical features of the superyacht, the locations of the anodes, and their characteristics. The shipyard designers selected the dimensions and the locations of the galvanic anodes according to their experience. All the potentials reported here refer to the Ag/AgCl reference electrode in artificial seawater (NaCl content 3.5%), which in such conditions has a potential of 0.26 V compared to the standard hydrogen electrode.

## 3. Results and Discussion

### 3.1. The Case Study: Traditional CP Design

**Figure 4:**
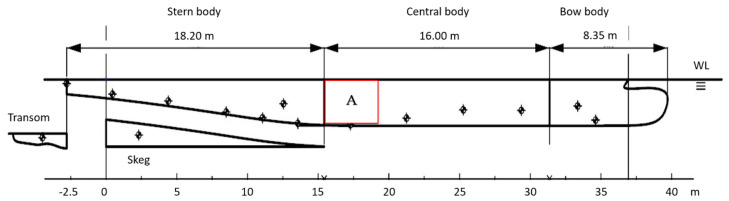
Hull subdivision for the calculation of anode masses. The red rectangular panel marked A is there to verify the reliability of the CP plan.

As a first step of the analysis, according to ISO 16222:2012, the hull was subdivided into five different bodies (Figure 4) to evaluate *I_c_* and then the anodic weight required. The five sections (from the left to the right of Figure 4) were: transom, skeg, stem body, central body, and bow body.

The CP plan allocates, for each anode, a surface to be protected. The anode in panel A is the most critical, because it protects the biggest portion of the submerged hull (*S* = 21.6 m^2^).

In Equation (4), *J_bd_* and *J_bs_* represent dynamic and static conditions, respectively. In accordance with the ISO and with the motoryacht’s top speed, it is reasonable to assume *J_bd_* = 500 mA/m^2^ and *J_bs_* = 100 mA/m^2^. Moreover, ships of this kind are usually in service for a limited period of the year, and for the rest of the time remain berthed. Based on this fact, a value of *χ* = 0.3, corresponding to navigation for 7 months in two years, is sufficient to estimate a conservative sailing period.

The following points are considered to evaluate the breakdown factor. The case study is a 42 m luxury motoryacht, characterized by a 500 micron epoxy protective coating composed of four coating layers covered with a 120 micron antifouling paint. For this type of ship, a thick layer of levelling filler is applied underneath the mentioned protective coating till half a meter below the waterline. This provides further protection from collision due to solids floating on the waterline. A person qualified in surface treatments according to FROSIO or NACE certifications performed rigorous inspections while the coating system was applied. With such a protective coating system and consolidated quality procedures, adopting an initial coating breakdown factor of 1% is reasonable.

The short time between two consecutive dry-docking (2 years average) and the high quality of the painting [19] suggest an overall *f_c_* equal to 2%.

Then, by Equation (4) and the values assumed above for the parameters, the current needed to protect section A of the central body is equal to 0.095 A.

The protection plan fixes a *FU* to 0.8, and by applying Equation (3), the weight to be installed is 2.66 kg, i.e., a little more than one-third of the real anode installed onboard. For the sake of safety and simplicity, the same anode size can be applied in all the hull portions. With the *FU* of 0.8, the theoretical anodic mass remaining at the end of service is around to 0.54 kg. The current supplied by the anode is determined by applying Ohm’s law, I = ΔU/R_a_, where the net driving voltage (ΔU) is the difference between the anode potential (−1.1 V) and the minimum cathodic protection level (−0.8 V), both against a Ag/AgCl reference electrode. The following formula has been used to evaluate the anodic resistance (Ra) based on ISO standard for the selected anode shape [14]:

(14) $$R_{a}=\frac{\rho }{2S}$$

where *ρ* is the environment resistivity (for seawater *ρ* = 0.23 Ω m) and *S* is the arithmetic mean of anode length (*L*) and width (*W*) in meters. In this case, a commercial 2.7 kg long flush anode has been adopted as reference. This anode has starting sizes of L = 290 mm and W = 53 mm. Then, we considered *FU* = 0.8, and as suggested in ISO standard, the final anode dimensions were *L_f_* = 276 mm and *W_f_* = 31mm, giving an area of 0.038 m^2^.

Now, all the terms are known, and the current supplied by the anode at the end of its lifetime is equal to 0.41 A. This value is well above the mean current protection of 0.095 A and also over the maximum current output *I_max_* (0.216 A) requested during dynamic conditions [14]:

(15) $$I_{max}=S\times f_{c\;}\times J_{bd}$$

In conclusion, the CP system based on 2.7 kg anodes likely protects the hull for the entire time it in operation.

As a matter of fact, the real CP plan works with 7 kg anodes, and the reasons why these oversized anodes have been used are actually unknown, but this discrepancy is often encountered in motoryacht designs.

Possible motivations that justify such a choice could be:

- A dry-docking period greater than the theoretical two years;
- Unexpected coating system damage;
- Higher anode consumption with respect to the expected theoretical *FU* = 0.8, due to loss of anode mass.

**Figure 5:**
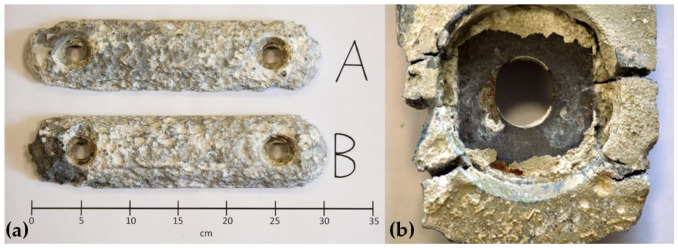
(**a**) Two end-of-life anodes (A) and (B) and (**b**) cracks of anode B in detail.

This last hypothesis was backed up by analyzing a bench of substituted anodes of the same origin as those installed from a similar case study motoryacht, as reported in Figure 5.

Nevertheless, only a 22% of weight loss could be estimated, the internal core was already visible, and several cricks and volume fractures were present in analyzed anodes. This evidence could somehow influence the design of the protection plan.

### 3.2. The Case Study: Numerical Simulation

The electrochemical parameters (exchange current densities, anodic and cathodic Tafel coefficient) required for the numerical analysis introduced in Section 2.3 have been extrapolated from potentiodynamic polarization measurements performed on the anode zinc alloy and the bare hull ship steel in artificial seawater. The values we obtained were i_0,a_ = 2 × 10^−4^ A cm^−2^, E_eq,a_ = −1.10 V vs. Ag/AgCl, a = 160 mV, i_0,c_ =1.48 × 10^−5^ A cm^−2^, E_eq,c_ = −0.60 V vs. Ag/AgCl, and b = 596 mV, and are in accordance with data available in the literature [20]. Figure S1 reports the mesh used to solve the equations system described in Section 2.3.

**Figure 6:**
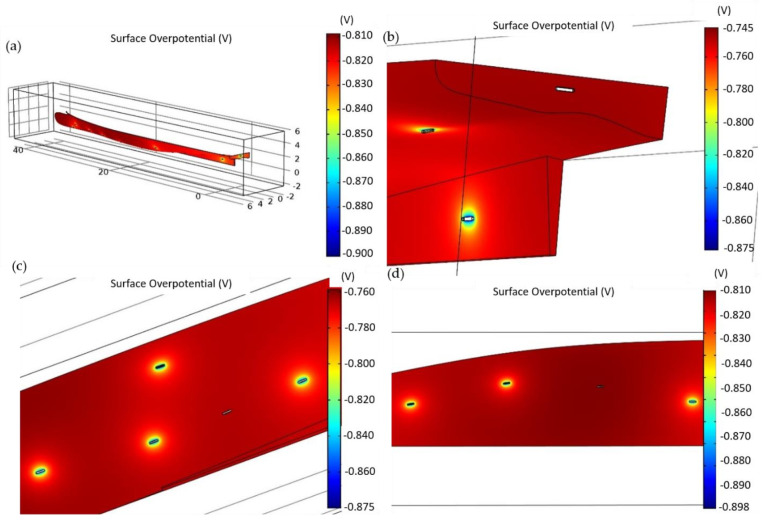
(**a**) Global view of the results of the numerical analysis with 2.7 kg anodes. (**b**–**d**) show the effect of deactivation of one anode in (**b**) transom, (**c**) stern body, (**d**) central body.

In Figure 6 the results of the numerical analysis are reported, in which we used 2.7 kg anodes. As can be seen, the maximum electric potential is −0.81 V, and so, according to the ISO Standard 16222:2012, the superyacht hull can be considered fully protected, providing all the anodes work properly.

Nevertheless, −0.81 V is extremely close to the threshold value of −0.80 V, and then a small variation in the breakdown factor due to coating damage, a different utilization factor, or the possible loss of one anode could be critical for the CP of the hull itself.

This lack of reliability is well reported in Figure 6b–d, which shows the effect of damages in a CP system, showing the overpotential variations when one anode is missing for three different sectors of the ship: (i) transom, (ii) stern, and (iii) central body, respectively. In the first two cases (Figure 5b,c), the loss of only one anode is sufficient to produce conditions out of protection (overpotential lower than −0.8 V). This means that the remaining 2.7 kg anodes are not able to provide protection for all surfaces. In the first case (transom body, Figure 6b), the protection loss is due to the fact that this surface does not host any anode, and the contribution of the current lines from the other sacrificial electrodes is not sufficient. In the latter (stern body, Figure 6c), the lacking protection is ascribable to the anode capacity and their displacement is not optimized to face this failure. The central body is the only section able to remain protected with the deactivation of one anode, as reported in Figure 6d. Indeed, the removal of one anode decreases the overpotential to extremely close to −0.8 V; the highest value is −0.801 V. This result suggests that the reliability of the cathodic protection system design based on the ISO standard is highly dependent on an accurate inspection and maintenance of the protection system. A singular instance of damage is sufficient to take the hull out of protection conditions.

**Figure 7:**
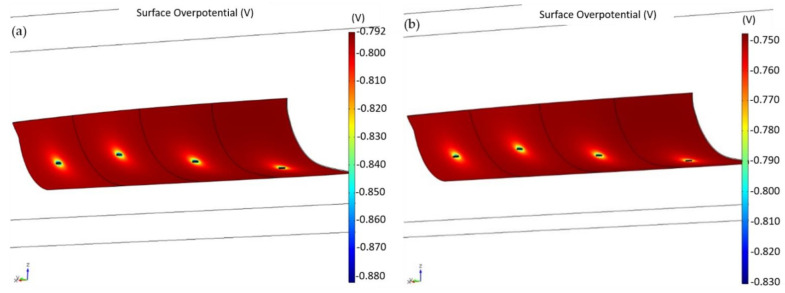
Results of the numerical analysis in the central body with (**a**) all 2.7 kg anodes at the beginning of their lifetimes and (**b**) all 2.7 kg anodes being 80% consumed (final weight 0.54 kg).

Figure 7a,b shows modelling results for the central body when all the anodes were active at the beginning of their lifetimes (2.7 kg) and after consumption of 80% (final weight 0.54 kg). The final configuration cannot protect the central body adequately because the geometry of the hull affects the potential distribution, which is not considered in Section 2.3.

These results give evidence for the necessity of interpreting ISO rules while considering strong safety factors.

**Figure 8:**
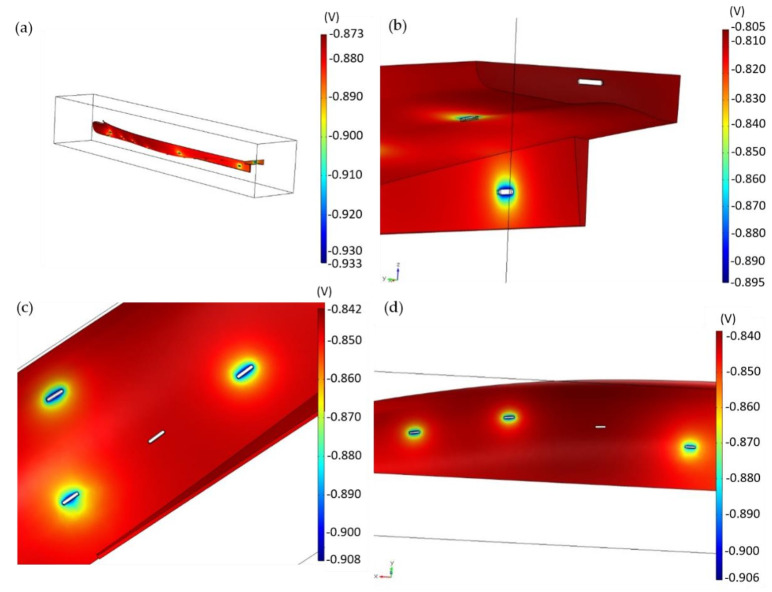
(**a**) Global view of the results of the numerical analysis with 7 kg anodes. (**b**–**d**) show the effect of deactivation of one anode in (**b**) transom, (**c**) stern body, (**d**) central body.

The following step of the numerical analysis considers the real anodic weight (7 kg) used for the cathodic protection system. In addition, in this case, analysis is presented for the full hull (Figure 8a) and for its main sections (Figure 8b–d) with damage to the CP system.

In Figure 8a, the superyacht in the reference condition appears to be more protected, since the maximum electric potential is −0.87 V. This potential is not too low as to endanger the hull to hydrogen evolution.

One-anode-missing situations were simulated for three sections, as reported by Figure 8b–d. As expected and discussed above for the case with 2.7 kg anodes, the transom body (Figure 8b) is more sensitive to the failure of the CP system. However, the lack of one anode is compensated by the adjacent electrodes, and the potential remains lower than the critical value of −0.8 V. The stern and central body sections better tolerate the shift from optimal conditions. In the stern body, where the ISO CP design is not able to compensate for the missing anode, the shipyard CP plan reaches a minimum value of −0.84 V, keeping the hull section in the safety zone. Similarly, in the central body the overpotential remains in an acceptable region, as already detected for the less reliable CP plan based on 2.7 kg anodes.

With the increased anodic weight of 7 kg, the unique off-design condition that does not guarantee a protected hull is the failure of two anodes in the transom body (Figure S2a,b). This is due to the geometry of the area. In the central body, the off-design condition still allowed proper cathodic protection, even in absence of two anodes (Figure S3a,b). This analysis brought to light the reasons for the choices of the shipyard in the design of the adopted cathodic protection plan.

Although the CP design that can be obtained through the ISO standard follows a reasonable selection of the different basic parameters, it highlights in remarkable vulnerability. However, the protection plan for this case study can be subjected to further optimization.

### 3.3. Investigation of the Effect of the Anodic Weight and Its Optimization

The investigation of a possible reduction in the anodic weight was performed considering panel A of Figure 4, which is the most critical, as introduced in Section 3.1.

**Figure 9:**
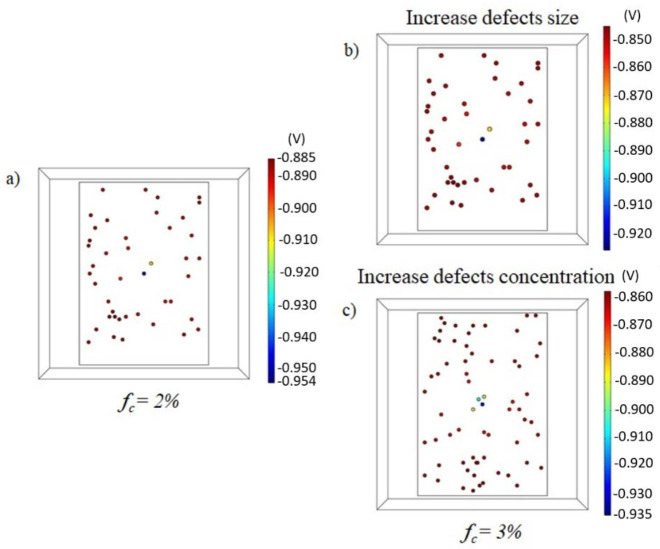
(**a**) Simulation of potential distribution on panel A with a 7 kg anode and coating defects equivalent to a fc = 2%. (**b**,**c**) Evaluation of potential distribution with fc = 3% considering an increase in (**b**) defect size or (**c**) defect concentration.

A MATLAB^®^ algorithm to obtain a random distribution of defects on the coated surface was coupled with the physics used for the numerical simulation. The algorithm, to simulate the lack of coating, provides center coordinates of all the stochastic defects. Figure 9a–c reports an example of an increase in uncoated metal starting from a situation of 2% uncoated metal (Figure 9a).

The increase in breakdown factor from 2 to 6% was investigated in two different situations:

(i) The number of defects (“concentration”) remained constant, and the size of each defect increased.
(ii) The size of each defects remained constant, and their concentration increased.

**Figure 10:**
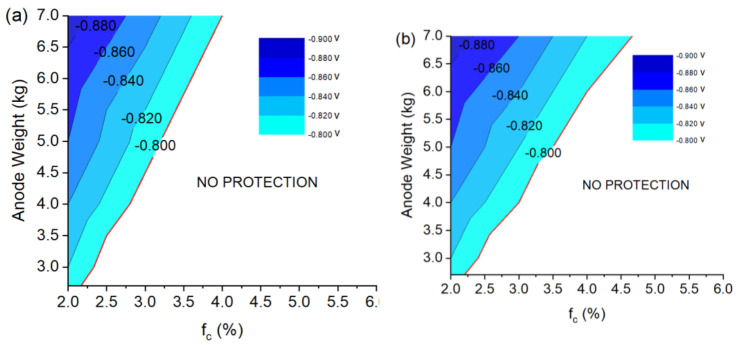
Minimum value of the protection overpotential as a function of breakdown factor and the anodic weight: (**a**) keeping constant the number of defects and increasing their size, (**b**) keeping constant the size of the defects and increasing their concentration.

Table 4 and the plots in Figure 10a,b summarize the minimum protection potential obtained in the two different cases listed above, considering the variation in the anode size from 2.7 to 7 kg.

As expected, the calculated values of the lower electrode potential in the selected panel as a function of the coating breakdown factor and anode weight are quite similar, and the results suggest two well-distinct points worthy of note:

- It is confirmed that the usage of a 2.7 kg anode, as proposed by the normative, provides protection with a limited safety margin;
- The 7 kg anode seems to be oversized, and a smaller electrode (5 kg) can already properly protect the surface for off-design conditions (an increase in breakdown factor and missing one anode) for the whole dry-docking period.

To confirm that the decrease in the anode weight from 7 to 5 kg is reasonable and guarantees a reliable safety margin, further simulations were performed on the whole hull with the suggested anode. Figure S4a supports the previous results, and installing 5 kg anodes on the hull surface has a minimum potential equal to −0.862, plenty above the threshold of −0.80 V. This choice keeps the hull completely protected while leaving a safety margin, even in off-design conditions, as reported in Figure S4b–d. The behavior is similar to that using 7 kg anodes.

## 4. Conclusions

In this paper, the cathodic protection by galvanic anodes for ships and superyachts has been analyzed, and a methodology to improve its application has been proposed.

To highlight the main features of this type of protection system, a real case study of a 42 m superyacht has been considered. The selection and analysis of the presented case study was performed on the basis of several real ships produced by different shipyards that presented similar characteristics. The first approach was based on the application of ISO design, which suggests the usage of 2.7 kg anodes.

This value is far from the 7 kg anodes installed on the real ship we considered. The possible reasons for this gap have been investigated by numerical analysis, which showed us the resilience of the real protection plan and evaluating the effects of hull sections out of protection in cases of excessive anode consumption or off-design conditions (e.g., lack of one or more anode).

This analysis highlighted that application of the guidance and rules that cover ship hull CP design can lead to limited safety margins if a critical approach to the estimation of the typical design parameters is not adopted.

On the other hand, further analysis has been devoted to the calculation of the minimum potential assumed in the most critical ship panel as a function of the coating breakdown factor and weight of the installed anode. The results suggest a feasible reduction in anodic mass from 7 to 5 kg. This decrease in the anode weight guaranteed the protection of the hull in off-design situations. Moreover, this new solution provides sufficient protection for the expected lifetime and preserves the system in cases of further damage to the hull and a consequent breakdown factor.

Despite the vast experience of professionals in CP design, the results reported in this paper well highlighted that there is space for new optimization tools and approaches not yet completely investigated, giving strength to the methodology proposed herein. The next steps of this research will be focused on extending the approach to other marine applications and to ICCP systems.

## Figures and Tables

**Table 1:** Differences between DNV.RP.B401 and ISO 16222:2012.

Factor	DNV.RP.B401	ISO 16222:2012
*J_b_*	*J_b_* = 100–170 mA/m^2^	*J_b_* = 100–500 mA/m^2^
*f_c_*	*f_c_* = k_1_ + k_2_t_design_depending on the draught and the coating thickness	*f_c_* = 0.5–3%
*FU*	*FU* = 0.8–0.9	*FU* = 0.7–0.95

**Table 2:** Geometrical features of the superyacht elected as a case study.

Length overall (L_OA_)	42.55 m
Length waterline (L_WL_)	39.78 m
Surface canoe body (S_cb_)	367.1 m^2^
Surface transom body (S_transom_)	4.3 m^2^
Surface skeg body (S_skeg_)	25.8 m^2^

**Table 3:** Characteristics of the anode used for the case study.

Weight	7 kg
Dimensions	455 × 90 × 40 mm
Material	Zn alloy
Reference potential	−1.10 (V vs. Ag|agCl)
Electric efficiency	0.95
Annual anodic consumption	C = 11.2 Ay/kg
Utilization factor	0.8
Design period	2 years

**Table 4:** Minimum protection potential obtained as a function of anode weight and breakdown factor: (a) keeping constant the number of defects and increasing their size, (b) keeping constant the size of defects and increasing their concentration. Below the red line the overpotential hull does not guarantee a complete protection.

(a)		**Anode Weight (kg)**					
		**2.7**	**3**	**4**	**5**	**6**	**7**
**Breakdown Factor (%)**	**2**	−0.81	−0.82	−0.84	−0.86	−0.87	−0.89
	**3**	−0.75	−0.76	−0.79	−0.81	−0.83	−0.85
	**4**	−0.70	−0.71	−0.74	−0.76	0.79	−0.81
	**5**	−0.68	−0.69	−0.71	−0.73	−0.76	−0.78
	**6**	−0.65	−0.66	−0.68	−0.71	−0.73	−0.75
(b)		**Anode Weight (kg)**					
		**2.7**	**3**	**4**	**5**	**6**	**7**
**Breakdown Factor (%)**	**2**	−0.81	−0.82	−0.84	−0.86	−0.87	−0.89
	**3**	−0.76	−0.77	−0.80	−0.82	−0.84	−0.86
	**4**	−0.72	−0.74	−0.75	−0.78	−0.80	−0.82
	**5**	−0.68	−0.70	−0.72	−0.75	−0.77	−0.79
	**6**	−0.66	−0.67	−0.70	−0.72	−0.75	−0.77

## Data Availability

The data presented in this study are available on request from the corresponding author.

## Supplementary Materials

The following supporting information can be downloaded at https://www.mdpi.com/article/10.3390/ma15072645/s1, Figure S1: Numerical mesh with tetrahedral elements; Figure S2: Effect of deactivation on hull potential of (a) two and (b) three anodes in the transom body with 7 kg anodes; Figure S3: Effect of deactivation on hull potential of (a) two and (b) three anodes in the stern body with 7 kg anodes. Figure S4: Global view of the results of the numerical analysis with 5 kg anodes. (b–d) show the effect of deactivation of one anode in (b) transom (c) stern body (d) central body.

## References

[1] Chen X., Li X.G., Du C.W., Cheng Y.F. (2009). Effect of cathodic protection on corrosion of pipeline steel under disbonded coating. Corros. Sci..

[2] Khambhaita P., Tighe-Ford D.J., Hinks K.J. (1995). Cathodic protection requirements of ship hull materials. Mater. Perform..

[3] Khoma M.S., Pokhmurs’kyi V.I. (2000). Corrosion-Fatigue Strength of Corrosion-Resistant Steels with Welded Joints. Mater. Sci..

[4] Lorenzi S., Pastore T., Bellezze T., Fratesi R. (2016). Cathodic protection modelling of a propeller shaft. Corros. Sci..

[5] Lebedev O., Menzilova M., Burmistrov E. (2021). Analysis of the use of paint coatings to protect the hull from corrosion. J. Phys. Conf. Ser..

[6] Ali A., Jamil M.I., Jiang J., Shoaib M., Amin B.U., Luo S., Zhan X., Chen F., Zhang Q. (2020). An overview of controlled-biocide-release coating based on polymer resin for marine antifouling applications. J. Polym. Res..

[7] Fayomi O.S.I., Agboola O., Akande I.G., Emmanuel A.O. (2020). Challenges of coatings in aerospace, automobile and marine industries. AIP Conf. Proc..

[8] Davies J., Truong-Ba H., Cholette M.E., Will G. (2021). Optimal inspections and maintenance planning for anti-corrosion coating failure on ships using non-homogeneous Poisson Processes. Ocean Eng..

[9] Xu L., Xin Y., Ma L., Zhang H., Lin Z., Li X. (2021). Challenges and solutions of cathodic protection for marine ships. Corros. Commun..

[10] Barbucci A., Bruzzone G., Delucchi M., Panizza M., Cerisola G. (2000). Breakdown of passivity of aluminium alloys by intermetallic phases in neutral chloride solution. Intermetallics.

[11] Barbucci A., Cerisola G., Bruzzone G., Saccone A. (1997). Activation of aluminium anodes by the presence of intermetallic compounds. Electrochim. Acta.

[12] Kalovelonis D.T., Rodopoulos D.C., Gortsas T.V., Polyzos D., Tsinopoulos S.V. (2020). Cathodic Protection of A Container Ship Using A Detailed BEM Model. J. Mar. Sci. Eng..

[13] Wang Y., KarisAllen K.J. (2017). Physical scale modelling of stray current interference to shipboard ICCP system. Corros. Eng. Sci. Technol..

[14] (2012). Cathodic Protection of Ship Hull.

[15] DNV (2010). Cathodic Protection Design.

[16] Gurrappa I., Yashwanth I.V.S., Mounika I. (2015). Cathodic Protection Technology for Protection of Naval Structures Against Corrosion. Proc. Natl. Acad. Sci. India Sect. A Phys. Sci..

[17] Dong C., Ji Y., Wei X., Xu A., Chen D., Li N., Kong D., Luo X., Xiao K., Li X. (2021). Integrated computation of corrosion: Modelling, simulation and applications. Corros. Commun..

[18] Bard A.J., Faulkner L.R. (1980). Electrochemical Methods: Fundamentals and Applications.

[19] Delucchi M., Ricotti R., Cerisola G. (2011). Influence of micro- and nano-fillers on chemico-physical properties of epoxy-based materials. Prog. Org. Coat..

[20] Kim J.-H., Kim Y.-S., Kim J.-G. (2016). Cathodic protection criteria of ship hull steel under flow condition in seawater. Ocean Eng..

