# Supplementary material for: A Critical Analysis on the Current Design Criteria for Cathodic Protection of Ships and Superyachts
Source: Materials (Basel). 2022 Apr 4;15(7):2645. doi: 10.3390/ma15072645 (PMC9000750; doi:10.3390/ma15072645)
Supplement: Supplementary file 1 [file materials-15-02645-s001.zip › materials-1650762-supplementary.pdf]

Supplementary Material

A Critical Analysis on the Current Design Criteria for Cathodic Protection of Ships and Superyachts

Davide Clematis [1,*], Alessandro Marroccu [2], Marco Panizza [1] and Antonio Barbucci [1,3]

[1] Department of Civil, Chemical and Environmental Engineering, University of Genova, 16145 Genova, Italy; marco.panizza@unige.it (M.P.); barbucci@unige.it (A.B.)
[2] Italy Operational Yachting Marine Surveyor, RINA Services S.p.A., Via Corsica 12, 16128 Genova, Italy; alessandro.marroccu@rina.org
[3] Institute of Condensed Matter Chemistry and Technology for Energy (ICMATE), National Research Council (CNR), c/o DICCA-UNIGE, Via All'Opera Pia 15, 16145 Genova, Italy
* Correspondence: davide.clematis@edu.unige.it

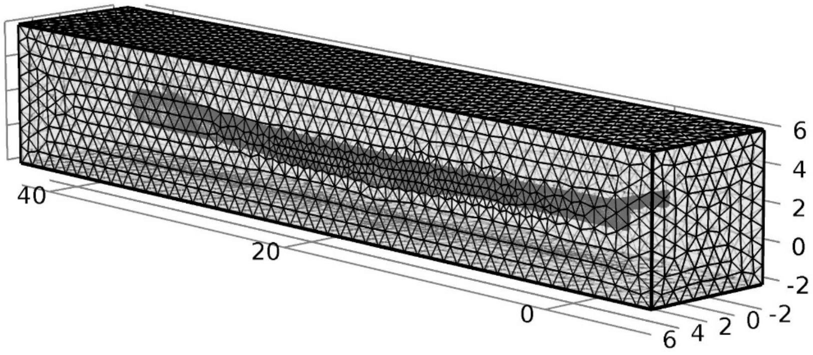

Figure S1. Numerical mesh with tetrahedral elements.

Simulations with 7 kg anodes

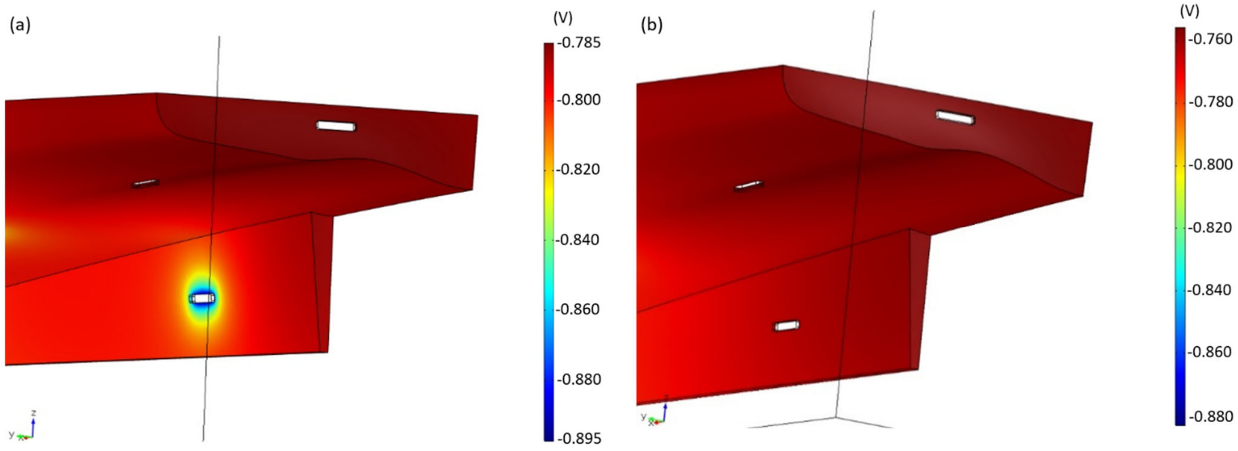

Figure S2. Effect of deactivation on hull potential of (**a**) two and (**b**) three anodes in the transom body with 7 kg anodes.

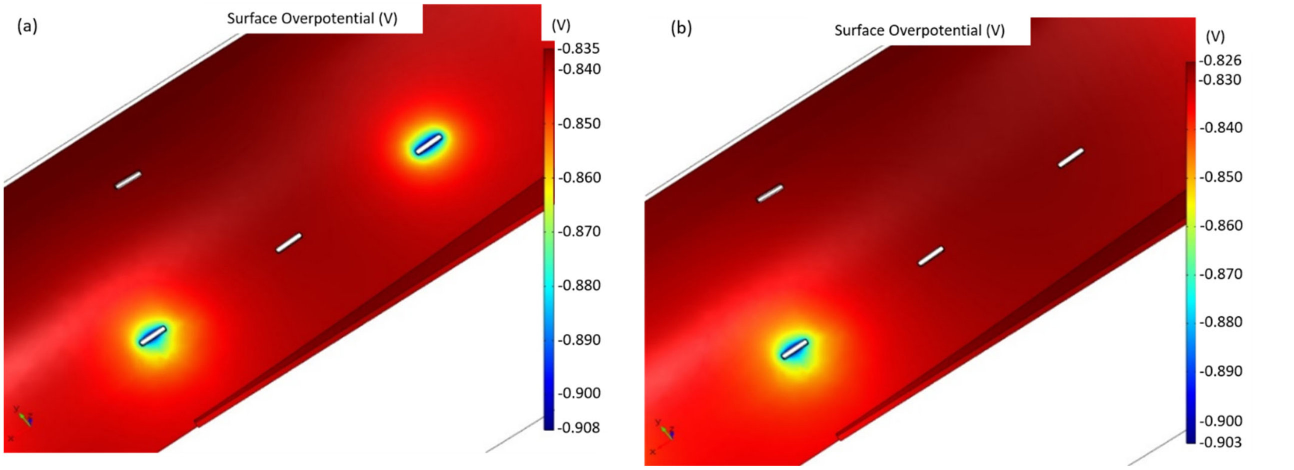

Figure S3. Effect of deactivation on hull potential of (**a**) two and (**b**) three anodes in the stern body with 7 kg anodes.

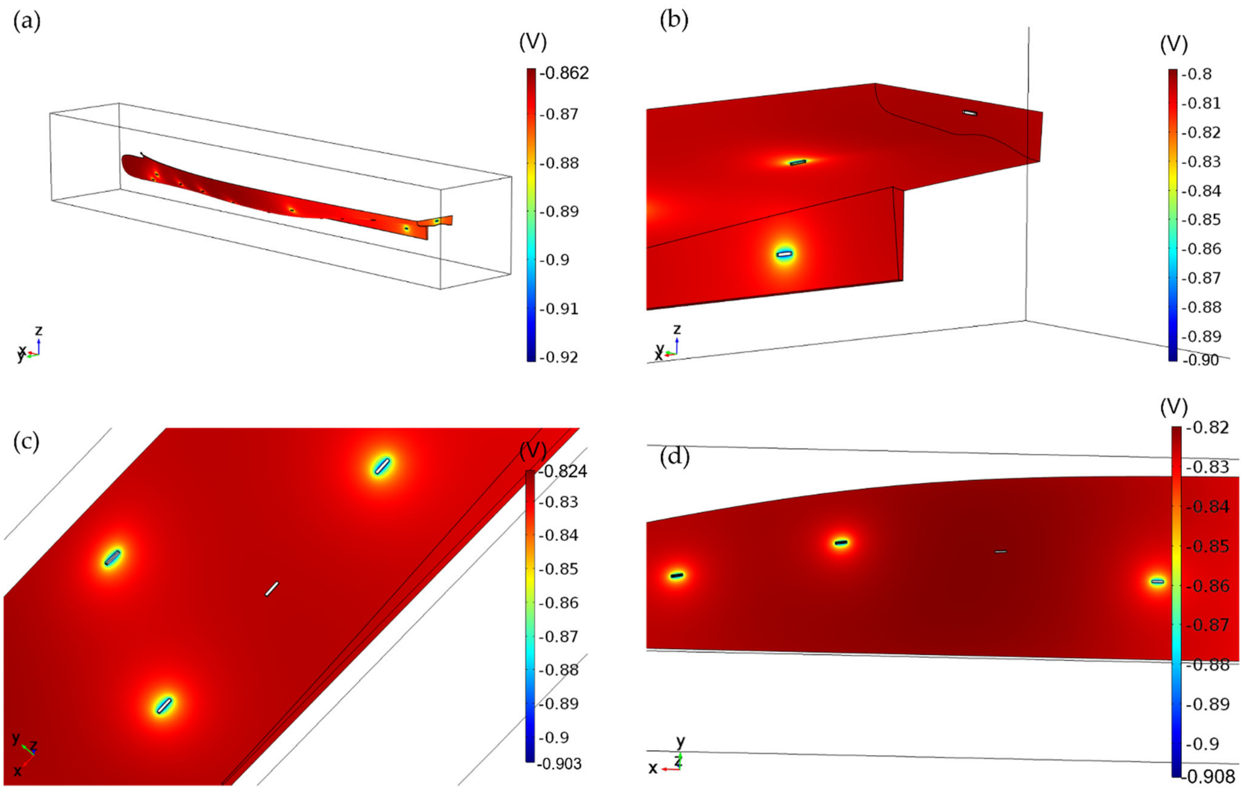

Figure S4. (**a**) Global view of the results of the numerical analysis with 5 kg anodes. Figures (**b**-**d**) show the effect of deactivation of one anode in (**b**) transom (**c**) stern body (**d**) central body.
